# Supplementary material for: Altered Blood–Brain Barrier Dynamics in the C9orf72 Hexanucleotide Repeat Expansion Mouse Model of Amyotrophic Lateral Sclerosis
Source: Pharmaceutics. 2022 Dec 14;14(12):2803. doi: 10.3390/pharmaceutics14122803 (PMC9783795; doi:10.3390/pharmaceutics14122803)
Supplement: Supplementary file 1 [file pharmaceutics-14-02803-s001.zip › pharmaceutics-2103819-supplementary figures.pdf]

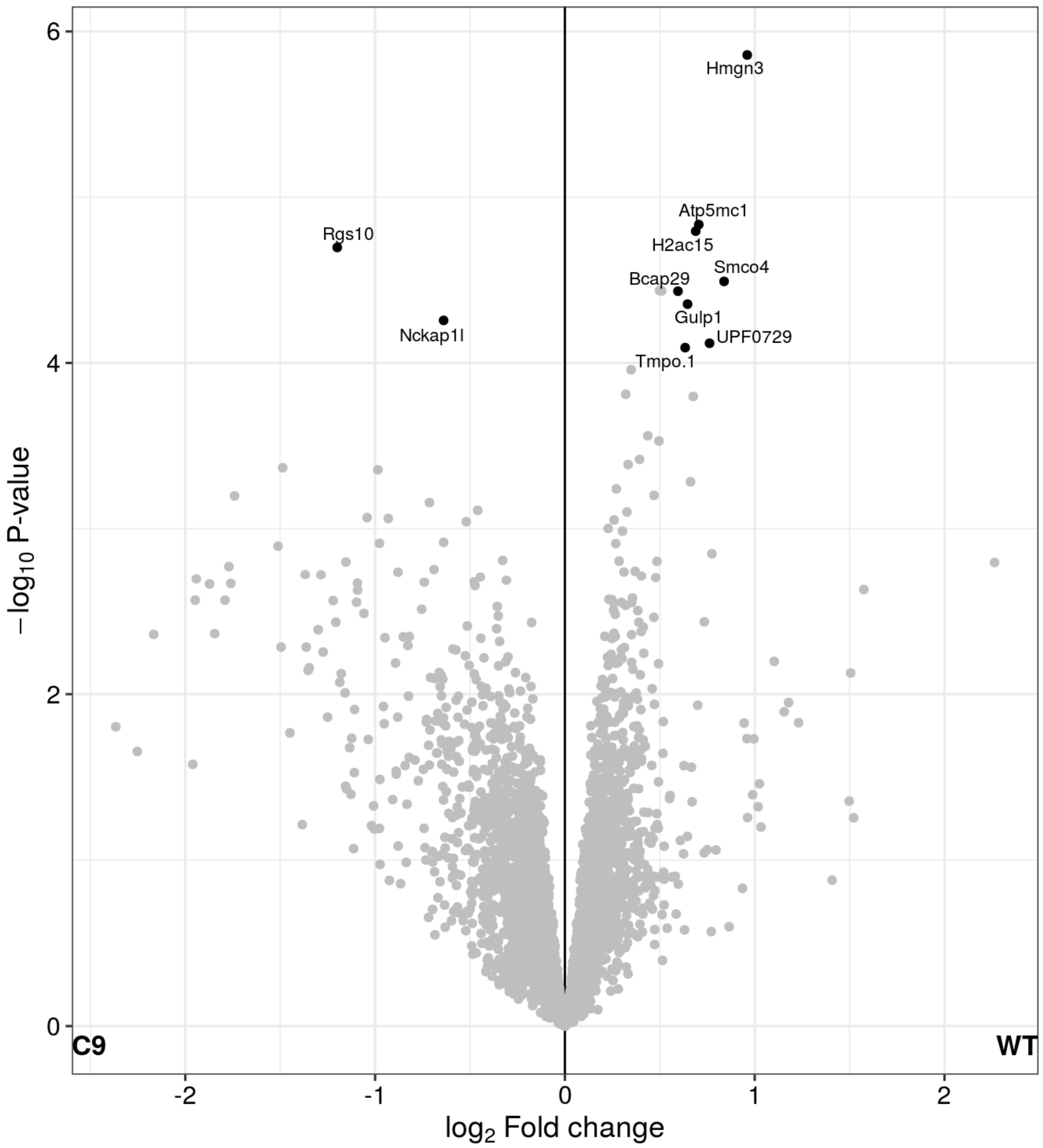

Figure S1. Volcano plot of differentially expressed proteins between = BMECs isolated from 145-150 days old female WT and C9 mice (n=8 per genotype).

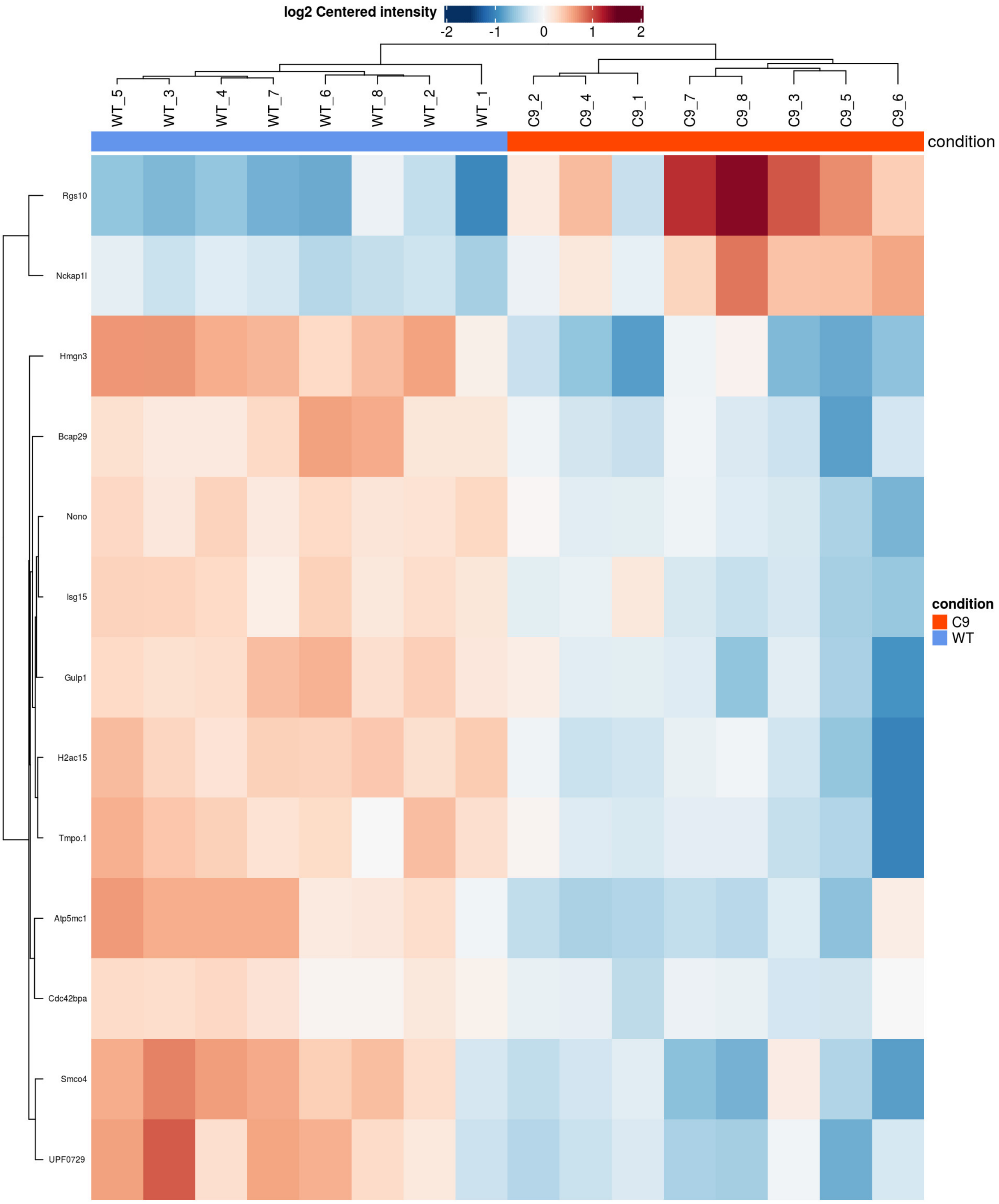

Figure S2. Heatmap of significant differentially expressed proteins between BMECs isolated from 145-150 day old female WT and C9 mice (n=8 per genotype).
